# Efficacy and safety of tafolecimab a new PCSK9 inhibitor in patients with hyperlipidemia: a systematic review and meta-analysis of randomized controlled trials

**DOI:** 10.1186/s43044-025-00653-z

**Published:** 2025-06-06

**Authors:** Ali Ashraf Salah Ahmed, Ahmad Alkheder, Mohamed Ahmed Ali, Mohamed R. Abdelraouf, Toka Ahmed Ashour, Hazem Mohamed Salamah, Abdelrahman Mahmoud, Diaa Hakim

**Affiliations:** 1 Faculty of Medicine, Minia University, Minia, Egypt; 2 Department of Otorhinolaryngology, Al Mouwasat University Hospital, Damascus University, Damascus, Syria; 3 Faculty of Medicine, Damascus University, Damascus, Syria; 4 Faculty of Medicine, Syrian Private University, Damascus, Syria; 5 Qena Faculty of Medicine, South Valley University, Qena, Egypt; 6 Faculty of Medicine, Alexandria University, Alexandria, Egypt; 7 Faculty of Medicine, Zagazig University, Zagazig, Egypt; 8 Brigham and Women’s Hospital, Harvard Medical School, Boston, USA; 9 Medical Research Group of Egypt (MRGE), Negida Academy, Arlington, MA USA

**Keywords:** Hyperlipidemia, Tafolecimab, Hypercholesterolemia, Hyperlipoproteinemia, Atherosclerosis

## Abstract

**Background:**

Hyperlipidemia is a common condition as nearly over 50% of adult Americans have high low-density lipoprotein (LDL) levels. Hyperlipidemia increases the risk of strokes, myocardial infarction, and other vascular events. PCSK9 monoclonal antibodies are one of the available options for the treatment of hyperlipidemia. Our study is a systematic review and meta-analysis that assesses the efficacy and safety of a new PCSK9 antibody, tafolecimab, in hyperlipidemia.

**Methods:**

Searching PubMed, EMBASE, Scopus, Web of Science (WOS), and Cochrane, we performed a PRISMA-based systematic review and meta-analysis to study effects of tafolecimab compared with placebo on different lipid indices that included LDL percent change, number of patients achieving ≥ 50% low-density lipoprotein cholesterol (LDL-C) reduction, LDL change from baseline, LDL change from baseline (CFB), apolipoprotein B CFB, and non-high-density lipoprotein cholesterol (non-HDL-C) CFB.

**Results:**

Four randomized controlled trials (RCTs) with 1093 patients were included in our study; 709 (64.87%) of them were males. Tafolecimab reduced LDL percent change [mean difference (MD) = − 62.28, 95% confidence interval (CI) (− 65.21, -59.35), *P* < 0.00001], the number of patients achieving ≥ 50% LDL-C reduction [MD = 46.92, 95% CI (21.91, 103.9), *P* < 0.0001], LDL CFB [MD = − 73.58, 95% CI (− 83.13, -64.03), *P* < 0.001], non-HDL-C CFB [MD = − 82.21, 95% CI (− 86.65, − 77.77), *P* < 0.001], apolipoprotein B CFB [MD = − 52.01, 95% CI (− 54.86, − 49.17, *P* < 0.001]. No difference was detected in overall adverse events (AEs) [risk ratio (RR) = 0.94, 95% CI (0.88, 1.01), *P* = 0.1128], serous AEs [RR = 0.93, 95% CI (0.57, 1.52), *P* = 0.7799], and AEs leading to treatment discontinuation [RR = 2.58, 95% CI (0.76, 8.77), but yielded more injection site reactions [RR = 2.53, 95% CI (1.14, 5.63), *P* = 0.0222].

**Conclusion:**

Tafolecimab is a valuable treatment option for hyperlipidemia, which showed improvement in several lipid indices (LDL, LDL-C, LDL CFB, non-HDL-C CFB, and apolipoprotein B CFB). However, it increased the rates of injection site reactions.

**Supplementary Information:**

The online version contains supplementary material available at 10.1186/s43044-025-00653-z.

## Background

Hyperlipidemia continues to be a serious health concern, which is defined as low-density lipoprotein (LDL), total cholesterol, triglyceride levels, or lipoprotein levels greater than the 90th percentile in comparison with the general population, or an HDL level less than the 10th percentile when compared to the general population [1], even though there have been some advancements in lipid-lowering therapies. As a matter of fact, a considerable number of patients still suffer from cardiovascular problems. Recently, new therapeutic modalities have emerged, of which tafolecimab, a monoclonal antibody that targets lipid metabolism pathways, got great emphasis because of its unique mode of action and promising preliminary results [2, 3]. Unlike other PCSK9 inhibitors requiring biweekly administration, tafolecimab demonstrates extended pharmacokinetic properties enabling monthly subcutaneous dosing while maintaining > 95% PCSK9 binding capacity throughout the dosing interval [4]. Early phase trials demonstrated robust LDL-C reduction at 12 weeks, results surpassing those observed with evolocumab in comparable populations [4]. Notably, post hoc analyses suggest potential pleiotropic benefits including reduced lipoprotein (a) levels and improved hs-CRP profiles, indicating broader atheroprotective effects [4].

Looking at hyperlipidemia management more closely, one can easily detect that traditional treatments sometimes fall short of the multiplicity in nature of the condition. Even though statins and other lipid-lowering medications have been shown to be safe and effective, their use is limited by muscle-related adverse events, new-onset diabetes, and hepatic toxicity, with 7–29% of patients exhibiting some form of statin intolerance [5]. Furthermore, their efficacy plateaus at higher doses due to compensatory PCSK9 upregulation, creating a therapeutic ceiling effect [5, 6], that require searching for alternative approaches. Statins have already been widely prescribed to be used for the initial treatment of hyperlipidemia, yet a large group of patients do not achieve their target low-density lipoprotein (LDL) levels even when taking the highest tolerated doses of statins and another group of patients showed resistance to statin therapy [6]. Given these limitations, there is a pressing need for alternative therapies that bypass compensatory mechanisms like PCSK9 upregulation, such as tafolecimab, a novel monoclonal antibody targeting PCSK9.

The mechanism of action of the drug tafolecimab is based on a specific type of inhibition of the enzyme proprotein convertase subtilisin/kexin type 9 (PCSK9), which is a key regulator of the degradation of the LDL receptor. Tafolecimab interacts with PCSK9 and consequently hinders its binding with LDL receptors which leads to an improved removal of LDL from the bloodstream and ultimately contributes to lowering the amount of LDL cholesterol in the blood. It is this mechanism’s precision that not only focuses on the underlying cause of hyperlipidemia but also appears to offer the opportunity for lipid profiles to be optimized as well as the prevention of cardiovascular events [6, 7].

Despite the randomized controlled trials (RCTs) conducted on tafolecimab and contributing to the existing evidence base, a comprehensive summary of available evidence through systematic review and meta-analysis is noticeably not widely discussed in the literature. This study aims to address the critical question: Does tafolecimab demonstrate superior efficacy and safety compared to placebo in reducing lipid parameters among patients with hyperlipidemia? Therefore, we thought of conducting this study to address this issue. In our current systematic review and meta-analysis, we strive to overcome this gap by carefully investigating tafolecimab efficacy and safety in hyperlipidemia patients using only RCTs.

## Methods

### Protocol registration

Our study was conducted based on the guidelines of the Preferred Reporting Items for Systematic Reviews and Meta-Analyses (PRISMA) statement [8], and the Cochrane Handbook of Systematic Reviews and Meta-analysis [9]. The pre-specified protocol was registered with PROSPERO on 26 April, 2024, with the ID: CRD42024536087.

### Data sources & search strategy

We systematically searched EMBASE, SCOPUS, Web of Science, MEDLINE (PubMed), and Cochrane Central Register of Controlled Trials databases until 29/3/2023. Searching databases was handled by two independent reviewers (A.A.S.A and A.M) without using filters. Table S1 shows the complete search strategy.

### Eligibility criteria

#### Inclusion criteria

Randomized controlled trials (RCTs) that matched our Population, Intervention, Comparator, and Outcomes (PICO) were included in this study. Our PICO criteria consist of population: patients with hyperlipidemia, intervention: tafolecimab, control: placebo, and outcomes: change in the LDL from baseline, patients achieving ≥ 50% LDL-C reduction from baseline, LDL percent change from baseline, apolipoprotein B change from baseline, non-HDL-C change from baseline, any adverse events (AEs), serious AEs, and AEs requiring intervention discontinuation. 

#### Exclusion criteria

We excluded non-RCT studies, such as in vitro studies, conference abstracts, observational studies, animal studies, and single-arm clinical trials.

### Study selection

Endnote software was used by two independent reviewers (T.A. and M.R.) for study selection. Duplicates were automatically eliminated by the software, and the remaining studies were screened by the reviewers using the title and abstract of each individual study. Studies that were included in the last step went through further screening using the full text of each study. Another reviewer (A.A.S.A) was called to settle the conflicts at each step of the screening.

### Data extraction

Two reviewers (T.A. and M.R.) independently used an Excel (2021 v17) extraction sheet that was generated after reading the full text of the included studies to extract the following needed data from the included RCTs. One sheet was a summary characteristics that included data about age, BMI, study design and phase, country where the study was done, and follow-up duration. Another sheet was baseline characteristics, which contained data about cardiovascular risk, concomitant disease, lipid-regulating medication N (%), and lipid parameters. The last two sheets contained data on efficacy outcomes and safety outcomes. More information about the extracted data is found in Tables 1 and 2.

**Table 1:** Summary characteristics

Study ID	Country	Study design and phase	Total participants	NCT	Type of hypercholesterolemia “Familial or Non”	Follow up duration	Dose
Chai et al. 2023 (13)	China	RCT Phase 3	148	NCT04179669	Heterozygous familial hypercholesterolemia	24 weeks	150 mg Q2W
							450 mg Q4W
Cui et al. 2021 (14)	China	RCT Phase1	30	NCT03366688	Hypercholesterolemia	14 Weeks	420 mg Q4W
							600 mg Q6W
Huo et al. 2023 (4)	China	RCT Phase 3	614	NCT04289285	Non-familial hypercholesterolemia	48 Weeks	450 mg Q4W
							600 mg Q6W
Qi et al. 2023 (15)	China	RCT Phase 3	303	NCT04709536	Heterozygous familial hypercholesterolemia	12 Weeks	450 mg Q4W

Study ID	Male N (%)		Age mean (SD)		BMI, kg/m2 mean (SD)	
	Tafolecimab	Placebo	Tafolecimab	Placebo	Tafolecimab	Placebo
Chai et al. 2023 (13)	29 (55.8)	11 (47.8)	48.7 (13.2)	48.0 (14.4)	N/A	N/A
	21 (43.8)	16 (64.0)	51.9 (10.7)	47.4 (14.2)	N/A	N/A
Cui et al. 2021 (14)	4 (50)	5 (42)	52.8 (13.60	44.3 (17.8)	23.0 (3.3)	24.9 (3.3)
	4 (50)	5 (42)	55.9 (9.8)	44.3 (17.8)	24.0 (1.9)	24.9 (3.3)
Huo et al. 2023 (4)	140 (67.0)	65 (64.4)	57.9 (9.03)	57.0 (9.15)	26.68 (3.68)	26.64 (3.33)
	133 (65.8)	67 (65.7)	57.7 (8.24)	57.0 (9.48)	26.62 (3.42)	26.66 (3.96)
Qi et al. 2023 (15)	147 (71.7)	62 (63.3)	56.9 ( 9.2)	56.8 (9.4)	26.6 (4.0)	26.3 ( 3.7)

N/A, not available; RCT, randomized controlled trial; SD, standard deviation; N, number, BMI, body mass index

**Table 2:** Baseline characteristics

Study ID	Cardiovascular risk				Concomitant disease									
	High		Very high		Cardiovascular disease		Cerebrovascular disease		Type 2 diabetes		Chronic kidney disease		Mixed dyslipidemia	
	Tafolecimab	Placebo	Tafolecimab	Placebo	Tafolecimab	Placebo	Tafolecimab	Placebo	Tafolecimab	Placebo	Tafolecimab	Placebo	Tafolecimab	Placebo
Chai et al. 2023 (13)	N/A	N/A	N/A	N/A	23 (44.2)	5 (21.7)	N/A	N/A	N/A	N/A	N/A	N/A	N/A	N/A
	N/A	N/A	N/A	N/A	19 (39.6)	9 (36.0)	N/A	N/A	N/A	N/A	N/A	N/A	N/A	N/A
Cui et al. 2021 (14)	N/A	N/A	N/A	N/A	N/A	N/A	N/A	N/A	N/A	N/A	N/A	N/A	N/A	N/A
	N/A	N/A	N/A	N/A	N/A	N/A	N/A	N/A	N/A	N/A	N/A	N/A	N/A	N/A
Huo et al. 2023 (4)	39 (18.7)	28 (27.7)	166 (79.4)	68 (67.3)	N/A	N/A	43 (20.6)	20 (19.8)	90 (43.1)	32 (31.7)	63 (30.1)	41 (40.6)	134 (64.1)	65 (64.4)
	45 (22.3)	24 (23.5)	143 (70.8)	70 (68.6)	N/A	N/A	47 (23.3)	19 (18.6)	68 (33.7)	35 (34.3)	74 (36.6)	34 (33.3)	115 (56.9)	64 (62.7)
Qi et al. 2023 (15)	54 (26.3)	23 (23.5)	150 (73.2)	75 (76.5)	105 (51.2)	52 (53.1)	44 (21.5)	16 (16.3)	68 (33.2)	36 (36.7)	70 (34.1)	31 (31.6)	113 (55.1)	57 (58.2)

Study ID	Lipid-regulating medication, N (%)						Lipid parameters			
	High-dose statin		Moderate-dose statin		Statin with ezetimibe		LDL-C, mg/dl mean (SD)		Non-HDL-C, mg/dl mean (SD)	
	Tafolecimab	Placebo	Tafolecimab	Placebo	Tafolecimab	Placebo	Tafolecimab	Placebo	Tafolecimab	Placebo
Chai et al. 2023 (13)	9 (17.3)	3 (13.0)	43 (82.7)	20 (87.0)	N/A	N/A	164.35 (42.92)	165.12 (37.90)	179.82 (49.50)	182.52 (42.54)
	4 (8.3)	2 (8.0)	43 (89.6)	23 (92.0)	N/A	N/A	162.80 (44.08)	167.05 (47.18)	177.88 (51.43)	183.68 (52.98)
Cui et al. 2021 (14)	N/A	N/A	N/A	N/A	N/A	N/A	150.81 (19.34)	146.95 (23.20)	N/A	N/A
	N/A	N/A	N/A	N/A	N/A	N/A	143.08 ( 23.20)	146.95 (23.20)	N/A	N/A
Huo et al. 2023 (4)	13 (6.2)	3 (3.0)	194 (92.8)	97 (96.0)	21 (10.0)	9(8.9)	109.44 (28.62)	109.44 (28.23)	120.65 (33.64)	120.65 (31.71)
	4 (2.0)	7 (6.9)	197 (97.5)	94 (92.2)	18 (8.9)	9(8.8)	112.53 (30.94)	109.05 (31.71)	123.36 (35.58)	121.42 (37.51)
Qi et al. 2023 (15)	7 (3.4)	1 (1.0)	198 (96.6)	97 (99.0)	N/A	N/A	117.94 (36.74)	119.88 (30.16)	134.96 (42.54)	136.12 (35.96)

N/A, not available; N, number; LDL, low-density lipoprotein; HDL, high-density lipoprotein

### Risk of bias assessment

Two reviewers (T.A. and M.R.) assessed the quality of the studies included in the research independently using the Cochrane ROB2 tool (the current version 22 August 2019) [10]. The ROB2 tool included five domains that were evaluated (the risk of bias resulting from the randomization process, the risk of bias due to deviation from the intended intervention, the risk of bias due to missing outcome data, the risk of bias in measuring the outcome, and the risk of bias in selecting the reported results). In case of any controversies, the reviewers discussed by explaining their point of view to each other and settled them via harmony. To estimate the quality of evidence, two reviewers (A.M. and H.M.S) utilized the Grading of Recommendations Assessment, Development, and Evaluation (GRADE) instructions [11, 12].

### Statistical analysis

The analysis was done with RevMan version 5.4. For dichotomous variables, we estimated the pooled risk ratio (RR) and 95% confidence interval (CI), and for continuous variables, we calculated mean differences with a 95% CI. The Chi-squared test and I^2^ were used to evaluate the statistical heterogeneity. When the heterogeneity was deemed significant (*p* 0.1 or I2 > 60%), we employed a random effects model; otherwise, we used a fixed effects model. We performed subgroup analysis based on the intensity and the type of used statin.

## Results

### Search results and study selection

**Fig. 1:**
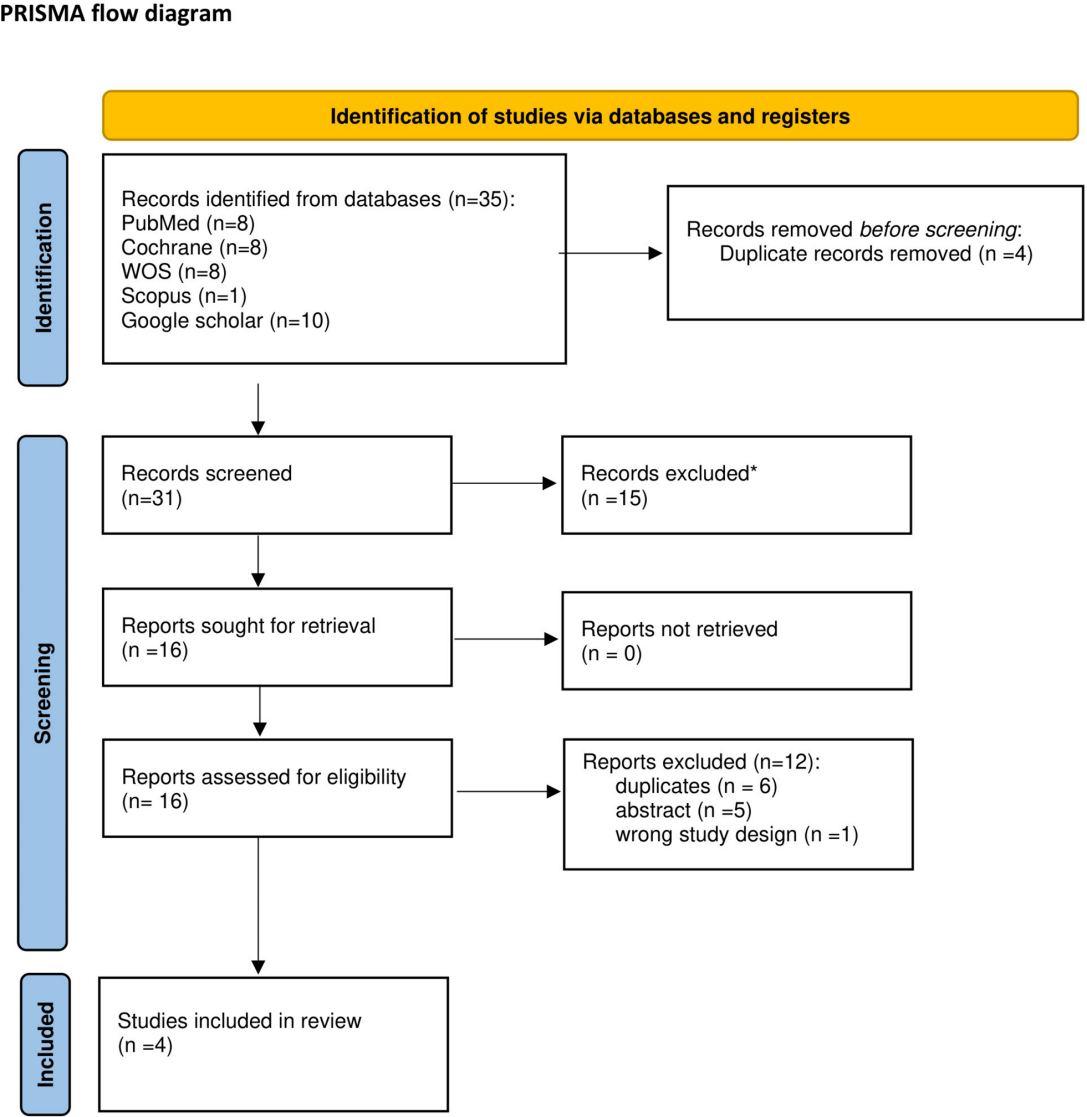
PRISMA flow chart of the screening process, which included searches of databases, registers, and other sources

A total of 35 studies were recognized and appraised for screening based on their titles and abstracts. After removing four replicas and 15 studies that did not meet the inclusion standards, 16 articles were evaluated by reading their full text. Out of these, four were eligible [4, 13–15] (Fig. 1).

### Characteristics of included studies

We incorporated four RCTs (4, 13–15) with 1093 patients; comprehensive summary and baseline information of the incorporated studies are listed in Tables 1 and 2.

### Risk of bias and quality of evidence

**Fig. 2:**
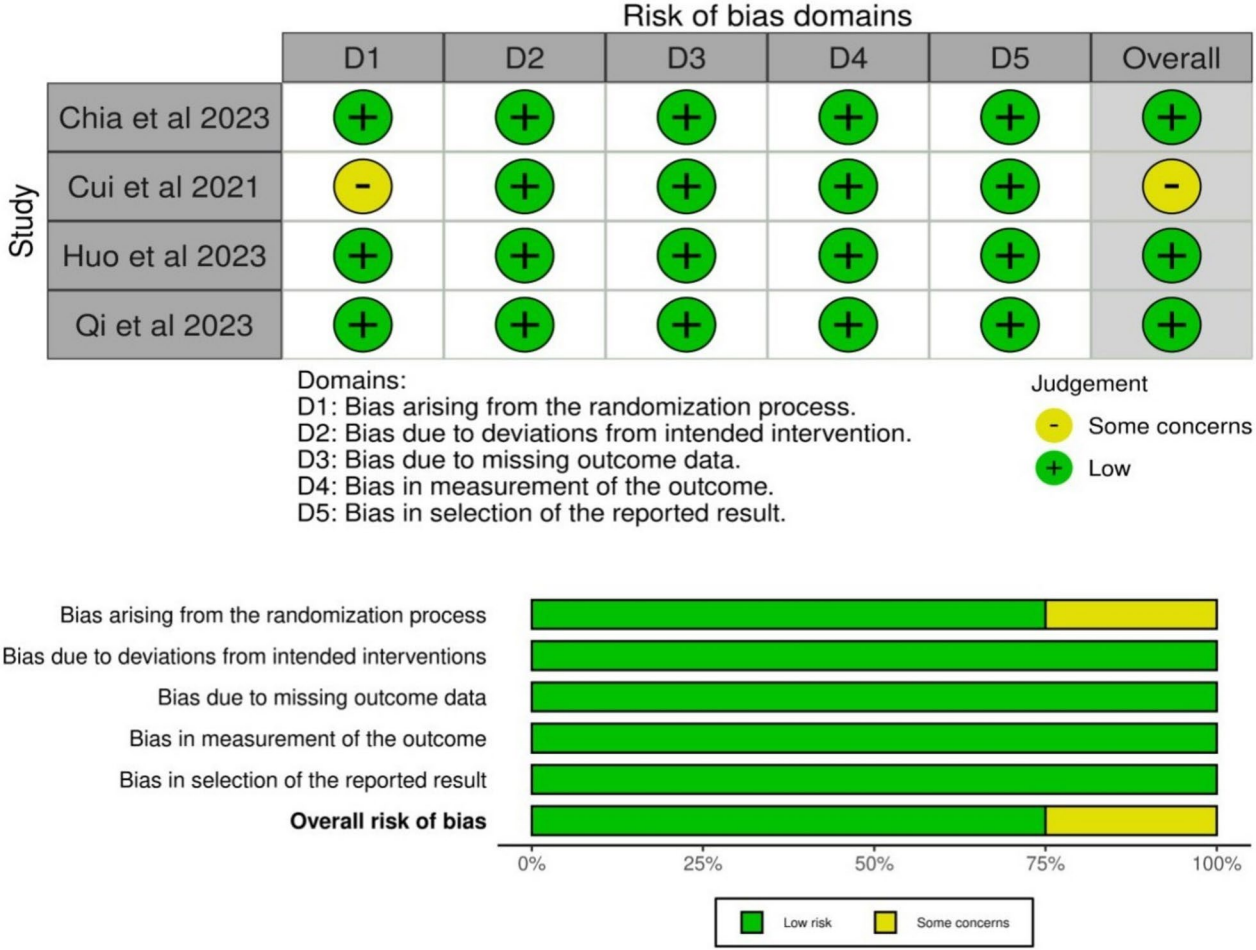
Summary of risk of bias (**A**—review authors’ judgments about each risk of bias item for each included study, **B**—review authors’ judgments about each risk of bias item presented as percentages across all included studies)

ROB 2.0 assessment showed that three studies had an overall low risk of bias [4, 13, 15], and one study showed some concerns [14]. More details are found in Fig. 2. The quality of evidence is illustrated in a GRADE-used profile (Table S2).

### Efficacy outcomes

#### LDL percent change

**Fig. 3:**
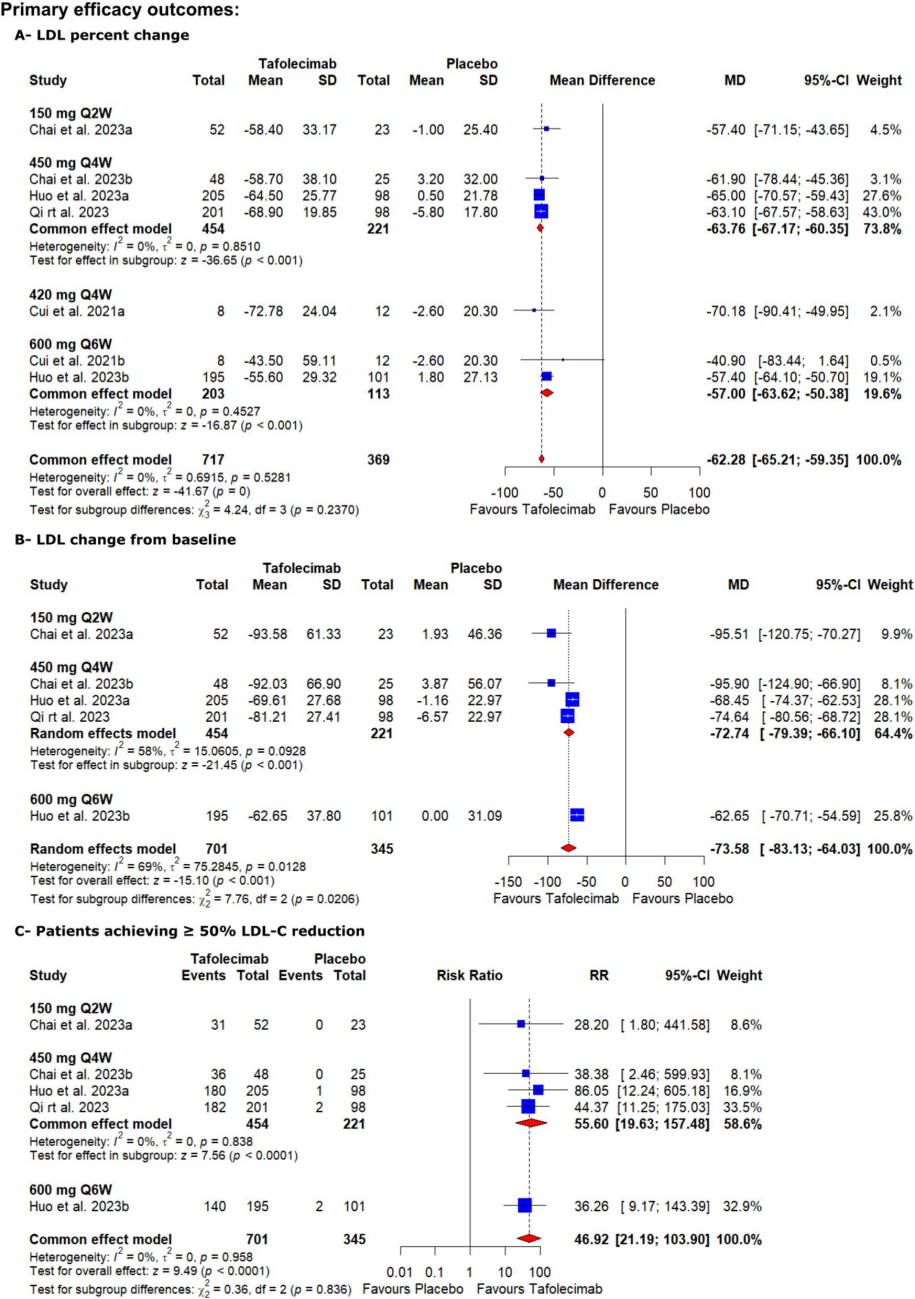
Forest plots show the mean difference in (**A**—LDL percent change from baseline, **B**—LDL change from baseline, and **C**—patients achieving ≥ 50% LDL-C reduction)

High-certainty evidence showed that tafolecimab significantly decreased LDL percent change from baseline level compared to the placebo [MD = − 62.28, 95% CI (− 65.21, − 59.35), *P* < 0.00001] (Table S2, Fig. 3). The pooled analysis was homogenous (I^2^ = 0%, *P* = 0.5281).

#### LDL change from baseline

Tafolecimab significantly decreased LDL level from baseline compared to the placebo [MD = − 73.58, 95% CI (− 83.13, − 64.03), *P* < 0.001] (Table S2, Fig. 3). The pooled analysis was heterogeneous (I^2^ = 69%, *P* = 0.0128), which could not be resolved via sensitivity analysis.

#### Patients achieving ≥ 50% LDL-C reduction

Similarly, the high-certainty evidence showed that tafolecimab significantly increased patients achieving ≥ 50% LDL-C reduction compared to the placebo [RR = 46.92, 95% CI (21.91, 103.9), *P* < 0.0001] (Table S2, Fig. 3). The pooled analysis was homogenous (I^2^ = 0%, *P* = 0.958).

#### Apolipoprotein B/apolipoprotein A1 change from baseline

**Fig. 4:**
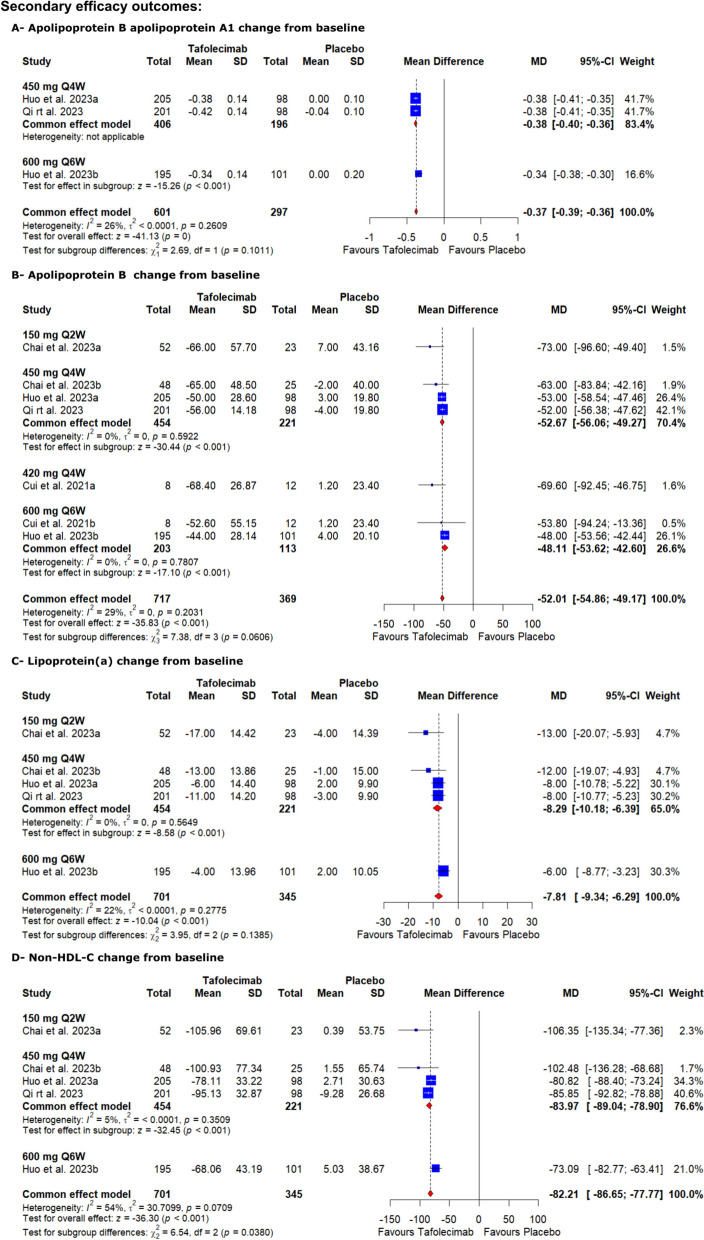
Forest plots show the mean difference in (**A**—apolipoprotein B/ apolipoprotein A1 change from baseline, **B**—apolipoprotein B change from baseline, **C**—lipoprotein(a) change from baseline, and **D**—non-HDL-C change from baseline)

As regards the Apolipoprotein B/ apolipoprotein A1, tafolecimab significantly decreased apolipoprotein B apolipoprotein A1 level from baseline compared to the placebo [MD = − 0.37, 95% CI (− 0.39, − 0.36), *P* < 0.001] (Table S2, Fig. 4). The pooled analysis was homogenous (I^2^ = 26%, *P* = 0.2609).

#### Apolipoprotein B change from baseline

On the other hand, tafolecimab significantly decreased apolipoprotein B level from baseline compared to the placebo [MD = − 52.01, 95% CI (− 54.86, − 49.17, *P* < 0.001] (Table S2, Fig. 4). The pooled analysis was homogenous (I^2^ = 29%, *P* = 0.2031).

#### Lipoprotein (a) change from baseline

High-certainty evidence showed that tafolecimab significantly decreased lipoprotein (a) change from baseline compared to the placebo [MD = − 7.81, 95% CI (− 9.34, − 6.29), *P* < 0.001] (Table S2, Fig. 4). The pooled analysis was homogenous (I^2^ = 22%, P = 0.2775).

#### Non-HDL-C change from baseline

Tafolecimab effect also extends to the non-HDL-C; high-certainty evidence showed that tafolecimab significantly decreased non-HDL-C change from baseline compared to the placebo [MD = − 82.21, 95% CI (− 86.65, − 77.77), *P* < 0.001] (Table S2, Fig. 4). The pooled analysis was heterogeneous (I^2^ = 54%, *P* = 0.0709), which could not be resolved via sensitivity analysis.

### Safety outcomes

#### Any adverse events

**Fig. 5:**
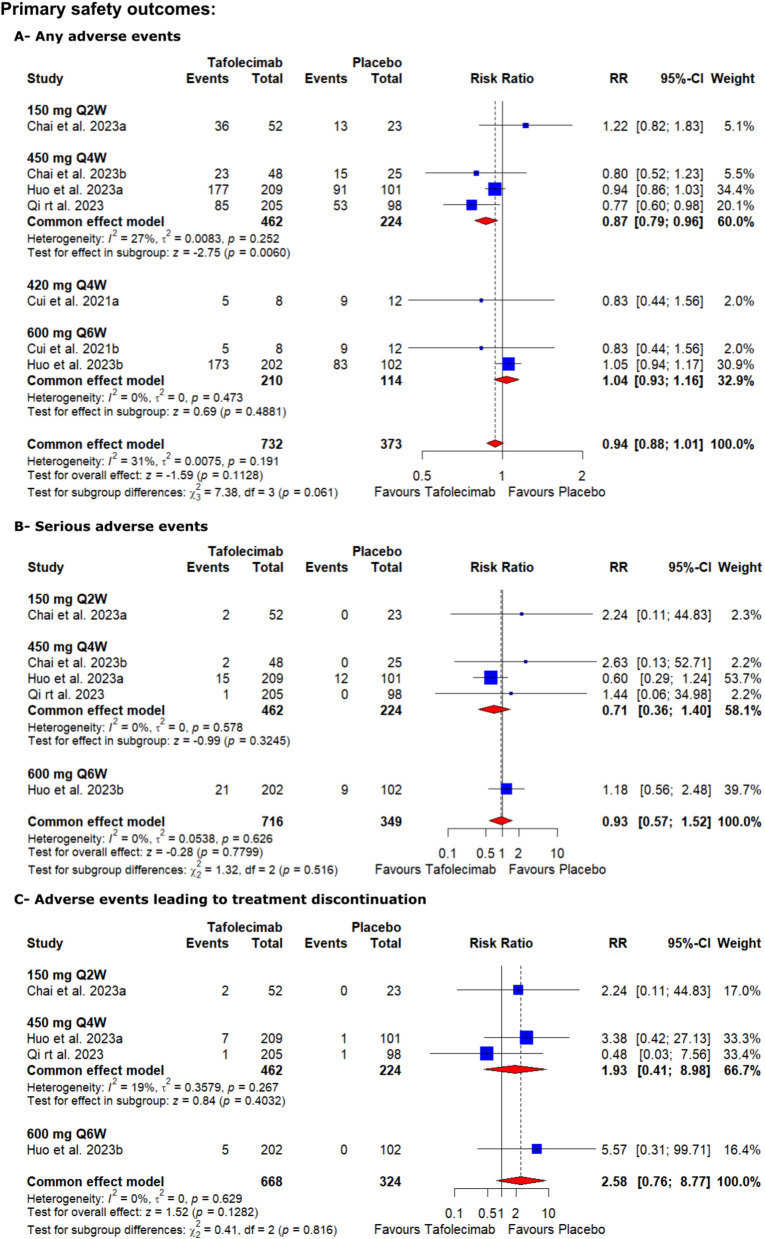
Forest plots show the risk ratio for the safety outcomes (**A**—any adverse events, **B**—serious adverse events, and **C**—adverse events leading to treatment discontinuation)

High-certainty evidence showed that tafolecimab non-significantly increased the risk of any adverse events compared to the placebo [RR = 0.94, 95% CI (0.88, 1.01), *P* = 0.1128] (Table S2, Fig. 5). The pooled analysis was homogenous (I^2^ = 31%, *P* = 0.191).

#### Serious adverse events

High-certainty evidence showed that tafolecimab non-significantly increased the risk of serious adverse events compared to the placebo [RR = 0.93, 95% CI (0.57, 1.52), *P* = 0.7799] (Table S2, Fig. 5). The pooled analysis was homogenous (I^2^ = 0%, *P* = 0.626).

#### Adverse events leading to treatment discontinuation

Low-certainty evidence showed that tafolecimab non-significantly increased the risk of adverse events leading to treatment discontinuation compared to the placebo [RR = 2.58, 95% CI (0.76, 8.77), *P* = 0.1282] (Table S2, Fig. 5). The pooled analysis was homogenous (I^2^ = 0%, *P* = 0.629).

#### Increased alanine aminotransferase

Moderate-certainty evidence showed that tafolecimab non-significantly increased the risk of increased alanine aminotransferase compared to the placebo [RR = 1.46, 95% CI (0.65, 3.3), *P* = 0.3582] (Table S2, Fig. S1). The pooled analysis was homogenous (I^2^ = 46%, *P* = 0.158).

#### Increased blood creatine phosphokinase

Moderate-certainty evidence showed that tafolecimab non-significantly increased the risk of increased blood creatine phosphokinase compared to the placebo [RR = 0.8, 95% CI (0.48, 1.32), *P* = 0.3827] (Table S2, Fig. S1). The pooled analysis was homogenous (I^2^ = 0%, *P* = 0.694).

#### Increased blood glucose

Low-certainty evidence showed that tafolecimab non-significantly increased the risk of increased blood glucose compared to the placebo [RR = 0.99, 95% CI (0.52, 1.88), *P* = 0.9722] (Table S2, Fig. S1). The pooled analysis was homogenous (I^2^ = 0%, *P* = 0.593).

#### Death

Low-certainty evidence showed that tafolecimab non-significantly increased risk of death compared to the placebo [RR = 0.68, 95% CI (0.13, 3.43), *P* = 0.641] (Table S2, Fig. S1). The pooled analysis was homogenous (I^2^ = 0%, *P* = 0.606).

#### Abnormal hepatic function

Moderate-certainty evidence showed that tafolecimab non-significantly increased the risk of abnormal hepatic function compared to the placebo [RR = 1.09, 95% CI (0.52, 2.26), *P* = 0.8272] (Table S2, Fig. S1). The pooled analysis was homogenous (I^2^ = 0%, *P* = 0.87).

#### Hypersensitivity

Moderate-certainty evidence showed that tafolecimab non-significantly increased the risk of hypersensitivity compared to the placebo [RR = 0.94, 95% CI (0.41, 2.41), *P* = 0.8843] (Table S2, Fig. S2). The pooled analysis was homogenous (I^2^ = 0%, *P* = 0.53).

#### Hyperuricemia

Low-certainty evidence showed that tafolecimab non-significantly increased the risk of hyperuricemia compared to the placebo [RR = 0.98, 95% CI (0.62, 1.55), *P* = 0.9345] (Table S2, Fig. S2). The pooled analysis was homogenous (I^2^ = 42%, *P* = 0.178).

#### Injection site reaction

Low-certainty evidence showed that tafolecimab significantly increased the risk of injection site reaction compared to the placebo [RR = 2.53, 95% CI (1.14, 5.63), *P* = 0.0222] (Table S2, Fig. S2). The pooled analysis was homogenous (I^2^ = 0%, *P* = 0.932).

#### Upper respiratory infection

Moderate-certainty evidence showed that tafolecimab non-significantly increased the risk of upper respiratory infection compared to the placebo [RR = 1.21, 95% CI (0.8, 1.81), *P* = 0.3648] (Table S2, Fig. S2). The pooled analysis was homogenous (I^2^ = 0%, *P* = 0.456).

#### Urinary tract infection

Low-certainty evidence showed that tafolecimab non-significantly increased the risk of urinary tract infection compared to the placebo [RR = 1.47, 95% CI (0.93, 2.32), *P* = 0.0986] (Table S2, Fig. S2). The pooled analysis was homogenous (I^2^ = 0%, *P* = 0.994).

## Discussion

Tafolecimab is a new member of the PCSK9 inhibitor family, a fully human immunoglobulin G2 (IgG2), developed as a treatment option in patients with hyperlipidemia [14].

Our systematic review and meta-analysis of four RCTs with 1093 patients provide robust evidence of tafolecimab’s efficacy in lowering lipid levels. Tafolecimab demonstrated significant reductions in LDL percent change, LDL change from baseline, the number of patients achieving ≥ 50% LDL-C reduction, non-HDL-C change from baseline, and apolipoprotein B change from baseline. These findings support tafolecimab’s role in hyperlipidemia management, particularly in patients with suboptimal response to traditional therapies.

These findings underscore the importance and advantages of tafolecimab as an addition to the therapeutic options to treat hyperlipidemia. Notably, the safety profile of tafolecimab was acceptable, with no significant difference detected in overall adverse events (AEs), serious AEs, and AEs leading to treatment discontinuation when compared to placebo. However, tafolecimab was associated with an increased risk of injection site reactions. This aspect necessitates careful consideration, especially in clinical practice, to balance the benefits of lipid reduction with potential adverse reactions.

Moreover, our analysis revealed other safety outcomes, including increased alanine aminotransferase, increased blood creatine phosphokinase, and abnormal hepatic function, although these findings did not reach statistical significance. These results highlight the importance of ongoing monitoring and further investigation into the long-term safety profile of tafolecimab.

The heterogeneity observed in the LDL change from baseline could not be resolved through sensitivity analysis, suggesting the influence of external factors or variations in study design. This aspect merits further investigation to elucidate the underlying causes and ensure the robustness of the efficacy outcomes.

Tafolecimab represents a promising addition to the armamentarium for hyperlipidemia management. Its mechanism of action, coupled with significant reductions in lipid levels observed in our analysis, positions it as a valuable therapeutic option. However, clinicians must remain vigilant regarding potential adverse reactions, particularly injection site reactions, and continue to monitor safety outcomes in clinical practice.

While PCSK9 inhibitors have been shown to be beneficial in a clinical setting, the high cost associated with these treatments curtails their widespread use. This highlights the importance of developing additional cost-effective medications within this class to improve availability and accessibility in healthcare systems [16–18].

In comparing the efficacy and safety profiles of tafolecimab with other PCSK9 inhibitors (alirocumab, evolocumab, and bococizumab). Firstly, in terms of effectiveness in lowering blood lipids, all drugs, including tafolecimab, demonstrated significant reductions in LDL cholesterol levels compared to placebos. Tafolecimab showed a robust reduction in LDL percent change (MD = − 62.28) and LDL change from baseline, along with an increase in the percentage of patients achieving ≥ 50% LDL-C reduction. Similarly, alirocumab, evolocumab, and bococizumab exhibited substantial reductions in LDL-C levels, albeit with varying magnitudes [19–22]. However, differences in efficacy exist between the drugs. For instance, while tafolecimab showed a significant decrease in non-HDL-C change from baseline, alirocumab, evolocumab, and bococizumab demonstrated comparable reductions in total cholesterol, triglycerides, and increases in HDL-C levels. Moreover, the potency of LDL-C reduction varied, with bococizumab exhibiting the most substantial decrease (− 56.96%) followed by evolocumab (− 53.99%) and alirocumab (− 51.29%), whereas tafolecimab showed a reduction in LDL levels from baseline of (− 62.28%) [19–22] according to our study. This difference in efficacy warrants further investigations and head-to-head comparisons between tafolecimab and other PCSK9 inhibitors. This observation could be due to different doses of the drugs and different time intervals between each of the doses.

Regarding safety, while all drugs exhibited tolerated or manageable adverse event, differences in specific adverse events were observed. Injection site reactions were significantly higher with tafolecimab compared to the other PCSK9 inhibitors, but this was judged as low quality of evidence by our GRADE system and should be interpreted with caution, warranting further investigation echoing findings from alirocumab, evolocumab, and bococizumab studies. However, myalgia incidence did not significantly differ between the drugs and their respective placebos [19–22].

In summary, while tafolecimab demonstrates efficacy and safety comparable to other PCSK9 inhibitors in patients with hyperlipidemia, subtle differences exist in their potency and specific adverse event profiles. Understanding these nuances can help in clinical decision-making regarding the selection of PCSK9 inhibitors based on patient characteristics and preferences.

### Strengths

This meta-analysis represents a comprehensive synthesis of evidence from four rigorously conducted randomized controlled trials, providing a robust assessment of tafolecimab’s efficacy and safety in hyperlipidemia management. Our meticulous approach, adhering to PRISMA guidelines and employing meta-regression, sensitivity, and subgroup analysis, enhances the reliability and validity of our findings. Additionally, our utilization of GRADE group recommendations ensures a thorough evaluation of the quality of evidence presented. Furthermore, our analysis encompasses a substantial number of patients from four randomized controlled trials (RCTs), enhancing the statistical power and generalizability of our findings.

### Limitations

Several limitations were considered. Heterogeneity observed in certain efficacy outcomes (e.g., LDL change from baseline) may introduce variability into pooled estimates, suggesting conclusions should be interpreted with caution. Although sensitivity analysis was conducted, the sources of heterogeneity could not be fully elucidated, suggesting potential confounding factors or variations in study design (might be related to the differences in the selected patient groups or comorbidities). Moreover, while our study focuses exclusively on RCTs to ensure methodological rigor, this limited the number of studies included; this approach may overlook valuable evidence from other study designs, such as observational studies or real-world data. Thus, our findings should be interpreted within the context of the available evidence landscape. Also, Cui et al. 2021 was a phase 1 RCT, which may limit its ability to evaluate the efficacy and safety of tafolecimab well [14].

### Implications for future research

Future studies should address several key areas to further enhance our understanding of tafolecimab’s role in hyperlipidemia management. Adherence to CONSORT reporting guidelines for clinical trials is imperative to enhance transparency and minimize reporting biases. Future longitudinal studies should include: (1) extended follow-up of existing trial cohorts (5–10 years) to assess sustained lipid-lowering effects and long-term safety; (2) real-world evidence analyses using national health databases to evaluate cardiovascular event prevention (e.g., stroke, myocardial infarction); and (3) cost-effectiveness studies comparing tafolecimab with other PCSK9 inhibitors over extended periods. Furthermore, comparative effectiveness studies comparing tafolecimab with other PCSK9 inhibitors, as well as traditional lipid-lowering therapies, can provide valuable insights into the relative efficacy and safety of different treatment options. Such studies can guide clinical decision-making and inform treatment guidelines for hyperlipidemia management. There is also a need to study the cost of this type of tafolecimab compared to other PCSK9 inhibitors.

## Conclusions

This study evaluated the efficacy and safety of tafolecimab in the management of hyperlipidemia. Tafolecimab was associated with significant reductions in LDL percent change, LDL change from baseline, and the number of patients achieving ≥ 50% LDL-C reduction, along with improvements in non-HDL-C and apolipoprotein B levels. However, tafolecimab was linked to a higher incidence of injection site reactions. Further research is needed to assess the long-term efficacy and safety profile of tafolecimab, as well as its cost-effectiveness.

## Supplementary Information

Additional file 1.

## Data Availability

Data and material are available through emailing the corresponding author.

## Abbreviations

LDL: Low-density lipoprotein
PCSK9: Proprotein convertase subtilisin/kexin type 9
LDL-C: Low-density lipoprotein cholesterol
Non-HDL-C: Non-high-density lipoprotein cholesterol
RCTs: Randomized controlled trials
PICO: Population, intervention, comparator, and outcomes
