# Supplementary material for: Efficacy and safety of tafolecimab a new PCSK9 inhibitor in patients with hyperlipidemia: a systematic review and meta-analysis of randomized controlled trials
Source: Egypt Heart J. 2025 Jun 6;77:56. doi: 10.1186/s43044-025-00653-z (PMC12144017; doi:10.1186/s43044-025-00653-z)
Supplement: Supplementary file 1 — Additional file 1. [file 43044_2025_653_MOESM1_ESM.docx]

**Contents:**

**Tables:
Table S1:** Search strategy.

**Table S2:** GRADE table results

**Figures:**

**Figure S1:** Forest plot of the secondary safety outcomes.

**Figure S2:** Forest plot of the continued secondary safety outcomes.

**Table S1:** search strategy. Date/ 29 / 3 /2024.

| Database | Search terms | Search field | Search results |
| --- | --- | --- | --- |
| Pubmed | (tafolecimab OR IBI 306) AND (hyperlipidemia OR hypercholesterolemia OR cholesterol* OR LDL OR non-HDL) | All Field | results  8 |
| Cochrane | (tafolecimab OR IBI 306) AND (hyperlipidemia OR hypercholesterolemia OR cholesterol* OR LDL OR non-HDL) | All Field | Trials  8 |
| WOS | (tafolecimab OR IBI 306) AND (hyperlipidemia OR hypercholesterolemia OR cholesterol* OR LDL OR non-HDL) | All Field | 8 |
| SCOPUS | (tafolecimab OR IBI 306) AND (hyperlipidemia OR hypercholesterolemia OR cholesterol* OR LDL OR non-HDL) | Title, Abstract, Keywords | Document results         1 |
| Google scholar | allintitle: Tafolecimab | Allintitle | 10 |

**Table S2:** GRADE results

| **Certainty assessment** | | | | | | | **Certainty** |
| --- | --- | --- | --- | --- | --- | --- | --- |
| **No of studies** | **Study design** | **Risk of bias** | **Inconsistency** | **Indirectness** | **Imprecision** | **Other considerations** |  |
| **LDL percent change** | | | | | | | |
| 4 | Randomized trials | Not serious | Not serious | Not serious | Not serious | Very strong association | ⨁⨁⨁⨁ High |
| **LDL percent change** | | | | | | | |
| 3 | Randomized trials | Not serious | Serious^a^ | Not serious | Not serious | Very strong association | ⨁⨁⨁⨁ High |
| **Non-HDL-C change from baseline** | | | | | | | |
| 3 | Randomized trials | Not serious | Not serious | Not serious | Not serious | Very strong association | ⨁⨁⨁⨁ High |
| **Apolipoprotein B change from baseline** | | | | | | | |
| 4 | Randomized trials | Not serious | Not serious | Not serious | Not serious | Very strong association | ⨁⨁⨁⨁ High |
| **Apolipoprotein B apolipoprotein A1 change from baseline** | | | | | | | |
| 2 | Randomized trials | Not serious | Not serious | Not serious | Not serious | None | ⨁⨁⨁⨁ High |
| **Lipoprotein(a) change from baseline** | | | | | | | |
| 3 | Randomized trials | Not serious | Not serious | Not serious | Not serious | None | ⨁⨁⨁⨁ High |
| **Patients achieving *≥* 50% reduction in LDL-C** | | | | | | | |
| 3 | Randomized trials | Not serious | Not serious | Not serious | Not serious | Very strong association | ⨁⨁⨁⨁ High |
| **Any adverse events** | | | | | | | |
| 4 | Randomized trials | Not serious | Not serious | Not serious | Not serious | None | ⨁⨁⨁⨁ High |
| **Adverse events Leading to treatment discontinuation** | | | | | | | |
| 3 | Randomized trials | Not serious | Not serious | Not serious | Very serious^b,c^ | None | ⨁⨁◯◯ Low |
| **Serious adverse events** | | | | | | | |
| 3 | Randomized trials | Not serious | Not serious | Not serious | Not serious | None | ⨁⨁⨁⨁ High |
| **Upper respiratory infection** | | | | | | | |
| 4 | Randomized trials | Not serious | Not serious | Not serious | Serious^b^ | None | ⨁⨁⨁◯ Moderate |
| **Injection site reaction** | | | | | | | |
| 3 | Randomized trials | Not serious | Not serious | Not serious | Very serious^b,c^ | None | ⨁⨁◯◯ Low |
| **Urinary tract infection** | | | | | | | |
| 2 | Randomized trials | Not serious | Not serious | Not serious | Very serious^b,c^ | None | ⨁⨁◯◯ Low |
| **Hypersensitivity** | | | | | | | |
| 3 | Randomized trials | Not serious | Not serious | Not serious | Serious^c^ | None | ⨁⨁⨁◯ Moderate |
| **Hyperuricemia** | | | | | | | |
| 2 | Randomized trials | Not serious | Not serious | Serious^d^ | Serious^c^ | None | ⨁⨁◯◯ Low |
| **Abnormal hepatic function** | | | | | | | |
| 2 | Randomized trials | Not serious | Not serious | Not serious | Serious^c^ | None | ⨁⨁⨁◯ Moderate |
| **Increased blood creatine phosphokinase** | | | | | | | |
| 2 | Randomized trials | Not serious | Not serious | Not serious | Serious^c^ | None | ⨁⨁⨁◯ Moderate |
| **Increased alanine aminotransferase** | | | | | | | |
| 2 | Randomized trials | Not serious | Not serious | Not serious | Serious^c^ | None | ⨁⨁⨁◯ Moderate |
| **Increased blood glucose** | | | | | | | |
| 1 | Randomized trials | Not serious | Not serious | Not serious | Very serious^c,e^ | None | ⨁⨁◯◯ Low |
| **Death** | | | | | | | |
| 2 | Randomized trials | Not serious | Not serious | Not serious | Very serious^f^ | None | ⨁⨁◯◯ Low |

**CI:** confidence interval; LDL; low density lipoprotein, HDL; high density lipoprotein.

**Explanations:**

a. High heterogeneity.

b. Wide CI.

c. small number of events.

d. Borderline significant difference between the different doses.

e. Evidence comes from only one study.

f. Very small number of events


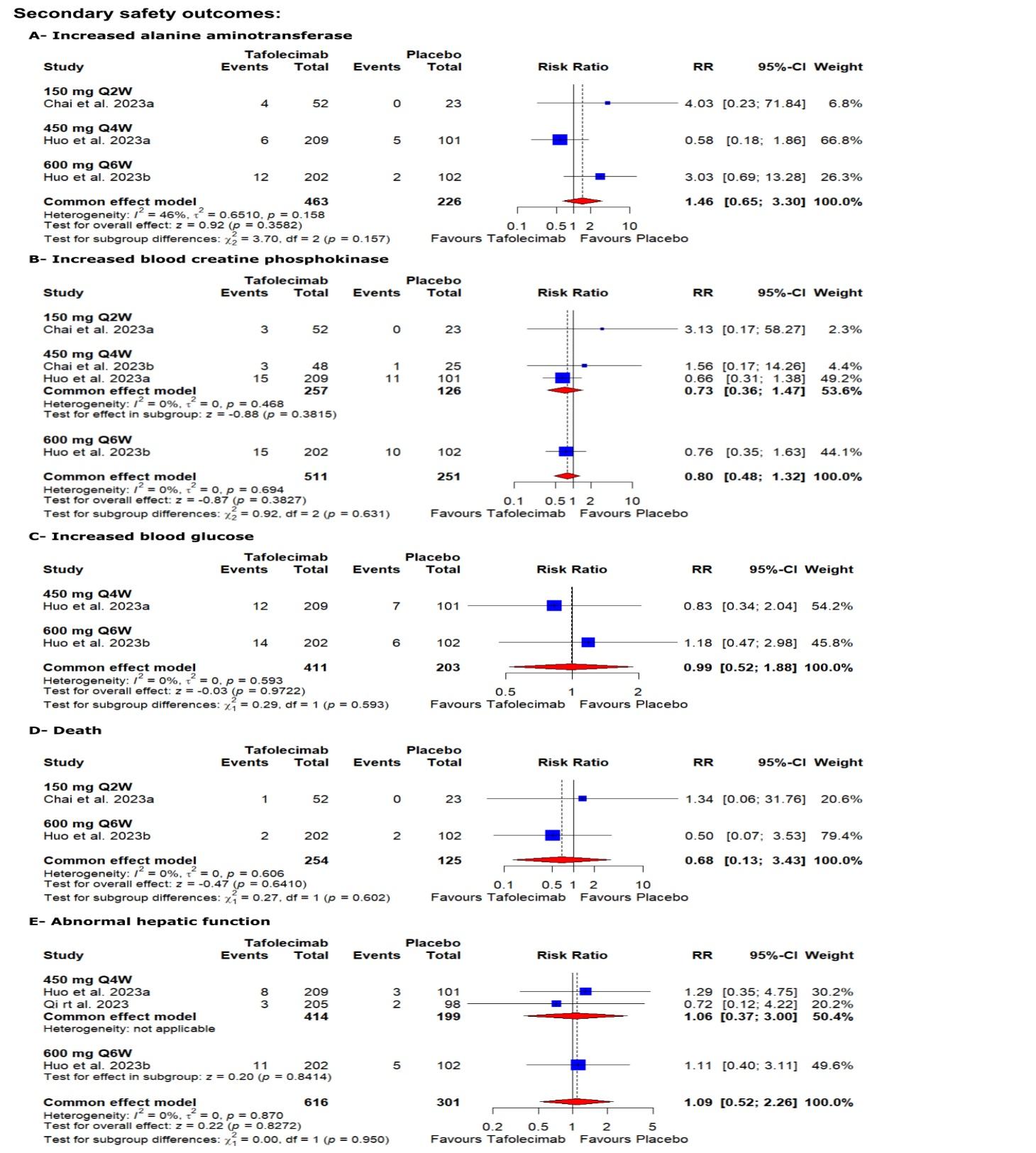


**Figure S1:** Forest plot of the secondary safety outcomes.


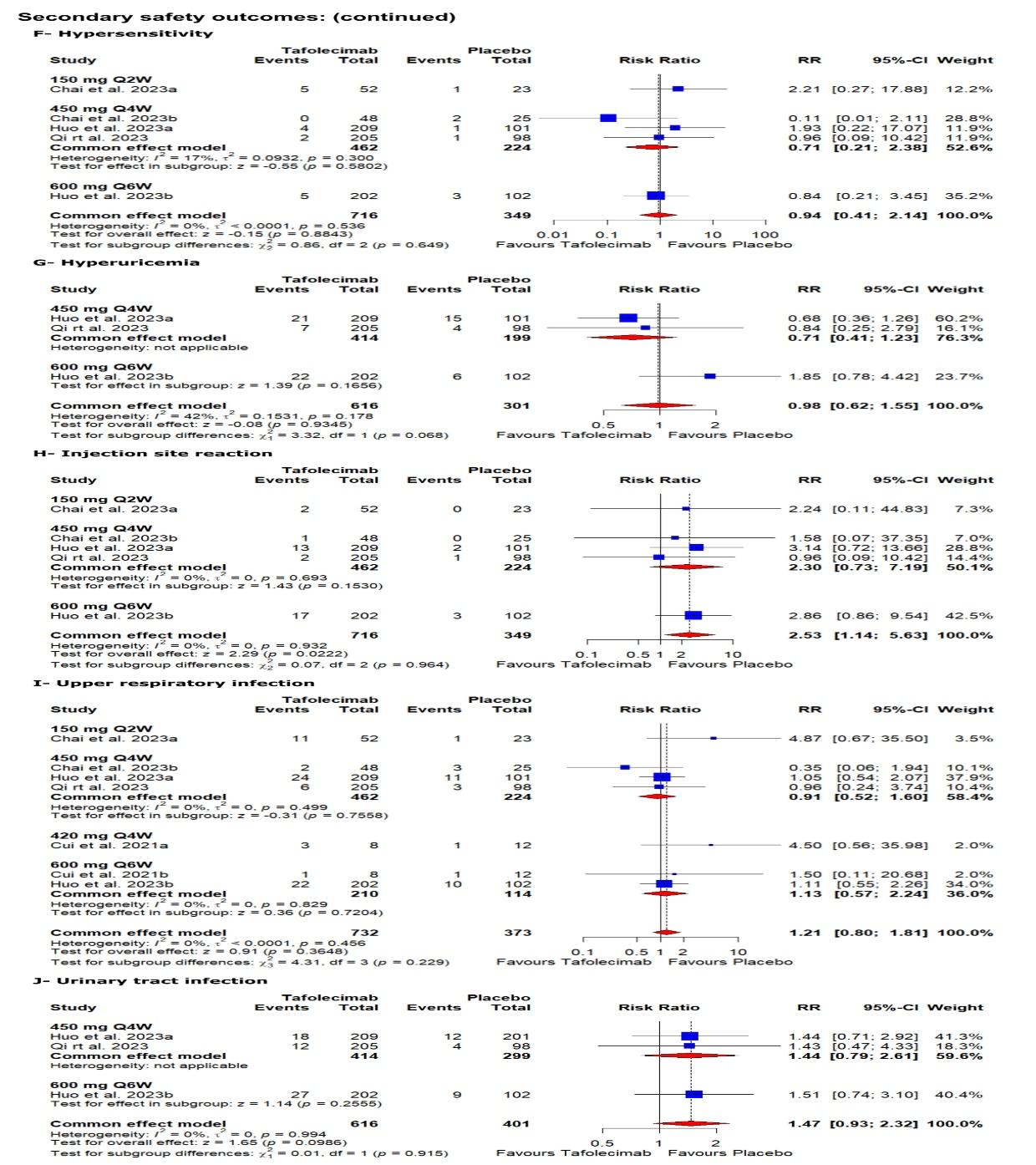


**Figure S2:** Forest plot of the continued secondary safety outcomes.
